# A study of liquidity cross-chain models based on convex optimization

**DOI:** 10.1038/s41598-024-78995-y

**Published:** 2024-11-28

**Authors:** Yutong Han, Chundong Wang, Huaibin Wang, Yi Yang, Xi Wang

**Affiliations:** 1 Tianjin Key Laboratory of Intelligence Computing and Novel Software Technology, Tianjin University of Technology, Tianjin, 300384 People’s Republic of China; 2 Ministry of Education, Key Laboratory of Computer Vision and System, Tianjin University of Technology, Tianjin, 300384 People’s Republic of China; 3 School of Computer Science and Engineering, Tianjin University of Technology, Tianjin, 300384 People’s Republic of China

**Keywords:** Blockchain, Convex optimization, Liquidity cross-chain, Contract pairs, Engineering, Mathematics and computing

## Abstract

Blockchain after several years of hustle, bustle, precipitation, and sublimation, with the landing application step by step towards maturity and development, gradually spawned the outreach needs of interacting with other applications. At this stage, only the mobility cross-chain interoperability technology is the infrastructure system that can perfectly solve such needs. To achieve a cross-chain system that defines and realizes user-free liquidity interoperability according to the cross-chain environment and cross-chain requirements, this paper designs a convex optimization-based liquidity contract pair cross-chain model. This model can realize a uniform distributed NFT ID construction identification method for the whole chain, and at the same time use the convex optimization model to obtain liquidity contract pairs that have and only have the global optimal matching, and in this way lock the shortest cross-chain time required, with AMM bonding curve management to achieve immediate liquidity effect, and realize the interconnection and interoperability of users’ assets with free liquidity. Finally, the time-consumption, robustness, and radius of convergence of the models are tested by comparing the model experiments and the proposed model’s proposed cross-chain incentive experiments yield results that verify that the proposed convex optimization-based liquidity contract is efficient, applicable, and secure for cross-chain models.

## Introduction

### Motivation

With the rapid development of the blockchain industry, the emergence of multiple public chains, private chains, and alliance chains creates a problem that how to communicate and even exchange values between different blockchains. To realize the autonomous and controllable organic integration of blockchain projects in different industries and achieve autonomous and controllable interconnection between blockchains, thus realizing the purpose of free flow of business and value between chains, blockchain has a practical and urgent need for mobile cross-chain interaction technology, and therefore, liquidity cross-chain technology arises^1,2^.

The mobility cross-chain model refers to the ability to allow digital information or value to flow freely and directly through the cross-chain model across the barriers between chains through certain technical means. The mobility cross-chain model has made breakthroughs in addressing blockchain interoperability, transactivity, and scalability from its generation, and development to practical application. The core element of the mobility cross-chain model is to help users on one chain exchange goods or information with users on another chain. From a business perspective, the liquidity cross-chain model is more like an intermediate exchange where users can go to the exchange to make cross-chain transactions. It is worth noting that a liquidity cross-chain transaction is an exchange of value, and it is important to ensure both the accuracy of the information flow and the reliability of the two-way value flow. Liquidity cross-chain technology is the bridge and hub to link the blockchain, the key to realizing the free interconnection of value, and a favorable means to expand the blockchain outward and break the value silos formed by the blockchain^3^.

The technical model for mobile cross-chain interaction can be either the notary model or the information lock model. The so-called notary model refers to the existence of a trusted notary node, which has the functions and powers of multiple-chain packing and sorting, in-chain blocking, etc^4^. Both parties across the chain submit their respective information to the notary, and in some cases, it is necessary to transfer all the information such as assets to the notary for verification, and the notary executes the exchange contract to exchange ownership, transfer exchange, and destroy/generate the information. This model is centralized, and the performance, security, and availability are completely dependent on the notary node. The so-called information locking model means that the initiator uses a puzzle and answer to lock the information and assets to be exchanged, specifying the recipient and restrictions such as time and block height. Within the restriction, the receiver can use the answer to extract the ownership of information, assets, etc. at any time^5–7^. If they are not extracted when the restriction is reached, the information and assets are returned to the initiator. Both parties involved in cross-chain can use this technology to complete information cross-chain. All in all, the improvement of liquidity cross-chain technology can be divided into the following four points.

- The underlying layer is lighter. the IBC protocol is touted as the gold standard for cross-chaining, but there can be some practical difficulties in implementing heterogeneous chains. The first is the transition from a repeater to a predictor. The main task of the repeater is to take the observed source chain information and commit it to the target chain, i.e., to complete the process of information transfer. Then, this process can be realized using a prophecy machine. That is, the prophecy machine changes from “down-chain to up-chain” to “up-chain to up-chain” information transfer. Secondly, the transition from the prophecy machine to TEE is to reduce the trust dependency on the prophecy machine, among which other technical means can be used to improve the work of blockhead synchronization. Finally, in the transition from Merkle Proof to ZK Proof, the current cross-chain implementation generally utilizes Merkle proof, but by generating zero-knowledge proof, it can also be used to solve the problem of the difficulty of the cost of signing in Ether, as a way to avoid the high-cost calculation associated with the execution of signatures in smart contracts.
- Better development support. From generic messages across chains to smart contract calls or from better multi-chain to full-chain SDKs.
- Innovation of liquidity in the application layer. AMM for shared liquidity, based on cross-chain protocols to enable management functions such as liquidity movement and settlement across chains.
- Consensus protocol improvement. Application chains are inadequate for maintaining their security and require certain cross-chain authentication methods to ensure mesh security.

Each blockchain has its own communication protocols, consensus rules, governance models, and native assets, and the core of liquidity across chains lies in automatic consensus^8^. That is, allowing one blockchain to have free access to the state of another blockchain facilitates the passing of information and assets between blockchains. However, liquidity cross-chain models are limited by security, interoperability, and decentralization. Therefore, the research focuses on improving the security of liquidity cross-chain models while also ensuring the interoperability of cross-chain models and resisting the impact of model decentralization.

### Research contributions

Liquidity cross-chain technology serves as a bridge connecting various blockchains, and its main application is to realize the functions of atomic free trading of assets, free interoperability of information, and complementary services between different blockchains. Currently, there are limitations because blockchain services in different domains are not deployed on the same blockchain underlying infrastructure, and existing projects and technologies cannot still freely communicate with each other between these infrastructures. In this paper, based on existing research, we propose a model architecture for liquidity contract pairs based on convex optimization as follows.

1. A chain-wide unified distributed autonomous cross-chain identity NFT is achieved.
2. Based on the characteristics of multiple scenarios with different liquidity requirements across chains, a convex optimization model is designed to obtain liquidity contract pairs with only globally optimal matching.
3. AMM combined with curve management for models based on liquidity contracts, to achieve the effect of free liquidity transfer of digital assets and free information interoperability to achieve instant liquidity.

### Paper structure

The rest of the manuscript is organized as follows: “Related work” section summarizes the representative existing cross-chain literature presents the existing problems of cross-chain models, and finally proposes the proposed system model of enhanced liquidity cross-chain in this paper. “Methods” section provides a new framework of the proposed liquidity cross-chain model, which unifies the NFT distributed autonomous cross-chain IDs to construct chain-wide identities, and the convex optimization-based liquidity contract pair model is also designed to enhance cross-chain liquidity. “Experiment results and analysis” section presents the experimental results of the proposed model. Finally, “Conclusion” section summarizes the work of this paper and briefly explains and analyzes the limitations of the work as well as the direction of future work.

## Related work

With the development of blockchain technology and the economy, the demand for data circulation and application collaboration among blockchains has become increasingly apparent^9^. The problems of throughput, scalability, and network isolation of blockchain itself restrict its further scale application, among which the isolation of the blockchain network is particularly prominent. Due to the differences in blockchain architecture, data structure, interface protocols, consensus mechanisms, and even business models, new “chain silos” have been formed among various blockchain applications, which directly limit the interoperability of asset exchange and business collaboration among them^10^. The difficulty of collaboration between different blockchain networks has limited the development of blockchain applications. Therefore, it is of great theoretical significance and practical value to study the breakthrough of universal liquidity cross-chain technology theory, solve the problem of liquidity cross-chain interoperability and multi-chain integration among blockchains, and realize safe and efficient data sharing and business collaboration among scenarios^11–13^. Table 1 demonstrates the nomenclature set of this paper.

Blockchain mobility cross-chain communication refers to allowing the free exchange of information assets between blockchains. The consensus body in the context of cross-chain communication determines how sure participants from one blockchain are of the state of a remote blockchain. Researchers usually enhance the development of blockchain cross-chain technology by building a federated blockchain together, unifying transaction system models, adapting consensus protocols/smart contracts, and prescribing authentication, to name a few. In the layout of the blockchain through the Coalition Blockchain, Jiang et al.^14^ propose a cross-chain framework to integrate multiple blockchains for efficient and secure IoT data management. It employs a federated blockchain as a control station and the solution establishes an interactive decentralized access model. Xiao et al.^15^ propose a federated blockchain extension architecture featuring integrated cross-chain functionality to provide a unified and trusted data-sharing infrastructure for open learning. In the layout of the UTS model, Kang et al.^16^ proposed efficient side-chain construction with fast cross-chain transport and demonstrated a small scale. Lei et al.^17^ proposes a cross-chain scheme for secure blockchain collaboration and interaction for efficient model training, model trading, and payments. In adjusting the layout of consensus protocols/smart contracts, Liu et al.^18^ proposed an improved method of blockchain cross-chain consensus algorithm based on weighted PBFT. Pang et al.^19^ proposed an architectural model for master-slave chain-based domain name resolution services, a multi-signature notary scheme to achieve cross-chain communication, and a PBFT algorithm based on the master-slave chain consensus algorithm (MSBFT). He et al.^20^ proposed a cross-chain trusted smart contract (C2T smart contract) to ensure the authenticity of cross-chain information, real-time and inter-chain write mutual exclusion, which makes the reputation calculation in the multi-chain billing model more convenient and accurate. In a layout that provides for authentication of identity, Chen et al.^21^ proposed a cross-domain authentication solution based on blockchain technology, which designed a blockchain trust model (BCBCA) and system architecture, designed a blockchain certificate by improving the X.509 certificate, developed a user cross-domain authentication protocol and re-authentication protocol process, and analyzed the security. The results show that the scheme has good scalability, security, and reliability, and can realize efficient and secure cross-domain authentication between domains. Shao et al.^22^ propose an identity-based encryption (IBE) IoT blockchain cross-chain communication mechanism (IBE-BCIOT). This mechanism selects proxy nodes of each blockchain among multiple blockchains and passes the IDs of the proxy nodes as public keys to the cross-chain notary. Then, the cross-chain notary office calculates the corresponding private key through the IBE mechanism and returns it to the proxy node in a secure way to achieve secure and efficient communication between blockchains. Next, the IBE-BCIOT mechanism provides two cross-chain communication schemes: the direct cross-chain communication scheme between proxy nodes, and the indirect cross-chain communication scheme through the notary. Table 2 compares the implementation methods of the current and proposed models as well as the target strategies.

**Table 1:** Nomenclature set.

Short title	Full name
IBC	Inter-blockchain communication
TEE	Trusted execution environment
SDK	Software development kit
NFT	Non-fungible token
AMM	Auto market maker
ERC	Ethereum request for comment
ETH	Ethereum

Liquidity cross-chain technology can reflect the liquidity of assets or transactions in the cross-chain process and is also an important indicator of the speed of the flow of transactions, assets, and other related information. However, since the liquidity cross-chain model is limited by the very different conditions of data structure, interface protocol, consensus mechanism, and even business model, it can only rely solely on the liquidity pool in the cross-chain model to provide liquidity cross-chain in a superficial sense. In research for liquidity cross-chain technology, researchers can only increase the liquidity of cross-chain model by single means such as prophecy machine, unified geographical authentication protocol, increasing cross-chain data and protocol security and privacy protection, improving cross-chain speed, changing cross-chain transaction model or optimizing the applicability of consensus algorithm, etc., without forming a spontaneous liquidity cross-chain chain model from the cross-chain asset or digital information itself liquidity cross-chain environment and cross-chain demand.

**Table 2:** Comparison of current and proposed models.

References	CA	SC	TS	AB	Identification	Liquidity
Jiang^14^				$\checkmark$		
Kang^16^			$\checkmark$			
Lei^17^			$\checkmark$			
Liu^18^	$\checkmark$					
Pang^19^	$\checkmark$					
Xiao^15^				$\checkmark$		
He^20^		$\checkmark$				
Hei^23^			$\checkmark$			
Chen^21^					$\checkmark$	
Shao^22^					$\checkmark$	
Su^24^			$\checkmark$			
Yin^25^		$\checkmark$				
Proposed model		$\checkmark$			$\checkmark$	$\checkmark$

*CA* consensus agreement, *SC* smart contracts, *TS* trading system model, *AB* alliance blockchain.

In summary, on the premise of ensuring a secure liquidity cross-chain model, this paper designs a liquidity contract pair cross-chain model based on convex optimization, unifies the distributed whole-chain autonomous cross-chain identity NFT, and realises the whole-chain NFT. At the same time, this cross-chain model can obtain the most matching liquidity contract pairs with AMM’s bonding curve management based on the convex optimization feature of obtaining the global optimal solution according to the cross-chain environment and cross-chain demand, to realize the free liquidity transfer of digital assets and free information interchange and achieve the effect of instant liquidity.

## Methods

In this section, based on the problem that liquidity contract pairs cannot match the best contract pairs according to the free liquidity cross-chain demand, we design a convex optimization-based liquidity contract pair model and a management model with AMM bonding curves. Firstly, in this paper, we set a unified distributed chain-wide autonomous cross-chain identity token, NFT, and the NFT data token stores and transmits data and value on blockchain networks such as Ether. It is then easier to access cross-chain service frameworks by generating NFT IDs. NFT-based smart contracts can be developed on different public chains, and they are not limited to any one public chain. In this paper, the public chains are demonstrated while the ERC standard is selected to develop NFT liquidity contract pairs.

**Fig. 1:**
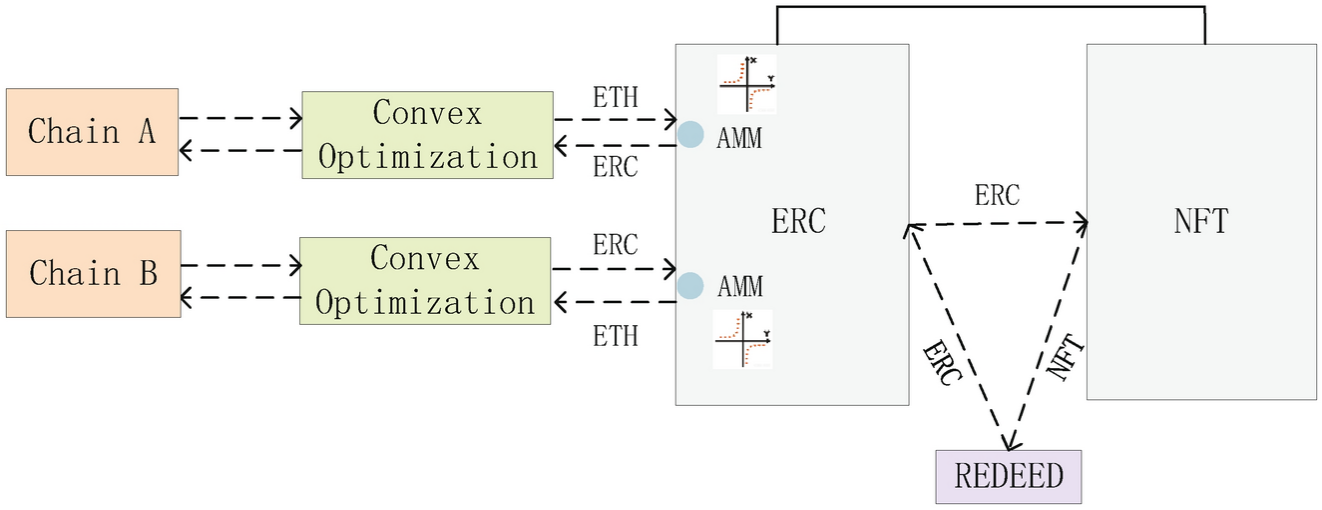
Convex optimisation based liquidity contract pair model.

Any artificial intelligence problem can be attributed to the combination of modeling and optimization. The model refers to the objective function, meaning the way to achieve the goal, and optimization is essentially training in machine learning to approach the goal more accurately and thus obtain the optimal solution. To reduce the cost time consumed by cross-chain, this paper collects liquidity cross-chain contract pairs of reward and time pairs. It is observed that the liquidity cross-chain contract pair reward then decreases as the time cost increases. Therefore, a convex curve is formed, which allows us to obtain the minimum-optimal point in the scheme set. That is, for the problem of optimal matching of liquidity contract pairs with the existence of contract pairs, convex optimization^26^ is the method that can perfectly solve this type of AI problem with only globally optimal solutions. The liquidity contract pair model based on convex optimization is shown in Fig. 1.

In a liquidity cross-chain system, after receiving a liquidity cross-chain task, the optimal liquidity contract pair is first obtained by analyzing the demand for the cross-chain scenario based on a convex optimization model, and then a liquidity contract pair is deployed, followed by the bonding curve management with AMM, allowing the cross-chain medium NFT to generate a trading market of its own so that NFT itself comes with liquidity and can be used without The NFT itself is liquid and can be cross-chained without an intermediary or regulator. This liquidity cross-chain model creates a new cross-chain model and enhances the value properties of the cross-chain medium itself. With this protocol, this means that anyone can conduct a cross-chain model at any time, reflecting instant liquidity, and the contract will adjust to the quantitative model requirements of the liquidity cross-chain value based on the supply in circulation. In the proposed model, after determining the autonomous liquidity cross-chain demand, the number of cross-chain and cross-chain expectations are calculated when the convex optimization-based liquidity contract pair is determined. The number of times a chain is crossed in the process directly affects the cross-chain reward, which affects the probability of occurrence of each contract pair at the same time.

In the proposed model, within a specified period after determining the demand for autonomous mobility across the chain, the system ranks the existing contract pairs for reward evaluation, and at the same time sends a reward evaluation signal to the existing contract pairs. When a contract pair receives a reward evaluation signal, the system collects data from all contract pairs forms a web of trust, and retains it. When the system does not receive an objection message within the specified time, the next step is performed. If there is a dissenting message, a re-award evaluation is sorted. Throughout the process, the system automatically collects all bonus evaluation history information and incorporates it into the new round of contract pair bonus evaluation ranking as the most up-to-date reference data. In each round, the system will reward the top 20% of pairs and eliminate the bottom pairs.

### Liquidity contract pairs

This paper is designed to implement blockchain liquidity replacement across blockchain technology using the concept of contract pairs^27,28^. According to the ERC specification, a smart contract can be written to create a “fungible pass”. A contract pair consisting of an ERC transaction contract and an ERC object contract is deployed through the protocol. The NFT is stateful, programmable, and networked, creating a graph in which objects, markets, and societies are all wrapped up. Each piece of information in the cross-chain identity becomes its micro-economy with native incentives for investment builders to develop spaces and systems for the ecology of objects. On the one hand, it allows the cross-chain of NFT to be decentralized, so that it can be truly mobile and instantly cross-chain; on the other hand, it allows NFT to conduct “fragmented” transactions, which lowers the threshold of NFT cross-chain. The dynamics and supply of ERC are managed by a bonding curve as AMM with instant liquidity, and the contract automatically adjusts the liquidity cross-chain credit based on the supply in circulation. This is a better way to implement a liquidity replacement cross-chain trading scenario. Generate the ERC algorithm as follows^29,30^.

Several constraint functions are required in the contract here. The first is the NFT holder, that is, the msg.sender(owner) and tokenId are one-to-many relationships, meaning that one person can own more than one NFT. Second, tokenId and tokenUrl are one-to-one relationships, representing a unique id on a chain per piece of data. There is no requirement that the tokenUrl be unique, but in the caller, the tokenUrl is usually set to be unique. It doesn’t matter if it’s not unique, the smaller the tokenId, the earlier it was set in the first place in the event of a conflict. Finally, after the NFT holder writes data to the chain, it can obtain the unique id of the NFT on the chain, and subsequent read and write operations can be conducted according to the id.

### Convex optimal liquidity contract pair mathematical model

First of all, in the liquidity cross-chain scenario, the reasonable matching of liquidity contract pairs will be affected by the liquidity cross-chain demand and there are many variations, so considering that the allocation of contract pairs with different demands in multiple scenarios can be considered as the solution of the convex optimization problem, the model is set uniformly as

1 $$\min \sum _{i=1}^N f_i\left( p_i\right) ,$$

2 $$1 \le p_i \le B_i (i \in \{1, 2,..., N\} ),$$

3 $$\sum _{i=1}^N p_i=P,$$

where, at a given liquidity cross-chain, the decision variables are divided into *N* groups, and *N* is the number of contract pairs in the cross-chain system; $f_i(p_i)$ is the objective function; the decision variable $p_i$ for the ith contract pair; $B_i$ denotes the priority of the ith contract pair; and *P* is the total number of resources.

In the set liquidity contract pairs cross-chain system, the objective of the liquidity cross-chain is to minimize the time cost for all users in the system, i.e. the objective function is considered a quadratic function

4 $$\begin{aligned} f_i\left( p_i\right) =A_i p_i^2-B_i p_i+C_i (i \in \{1, 2,..., N\}), \end{aligned}$$

where $A_i$, $B_i$, $C_i$ are the coefficients of the corresponding functions of the ith contract pair, respectively, $A_i$ denotes the liquidity cross-chain power consumption of the ith contract pair, $B_i$ denotes the liquidity cross-chain priority of the ith contract pair, and $C_i$ denotes the liquidity cross-chain complexity of the ith contract pair.

Second, to find the minimum cross-chain time corresponding to the reward of a rated liquidity cross-chain contract pair, a Bernoulli implementation is used. We assume that the probability of reaching liquidity cross-chain without regulation for the first time is $q_i$, in this case, will the liquidity cross-chain continue to be achieved in the same way as Bernoulli’s, i.e., the probability of reaching liquidity cross-chain for the second time is $(1 - {q_i}){q_i}$, and the probability of reaching liquidity cross-chain for the third time is ${(1 - {q_i})^2}{q_i}$, when by the $\xi$th day cross-chain, the liquidity cross-chain reward can be obtained as follows

5 $$\begin{aligned} q_i+\left( 1-q_i\right) q_i+\left( 1-q_i\right) ^2 q_i+\cdots +\left( 1-q_i\right) ^{\xi } q_i=1-\left( 1-q_i\right) ^{\xi +1}. \end{aligned}$$

Therefore, the expected number of liquidity cross-chain counts can be obtained by summing all the cross-chain count reward values. Aggregating the expected values of liquidity cross-chaining allows for determining the pair with the lowest reward and cost time.

Correspondingly, the Lagrange multiplier method needs to be used in solving the optimal solution to obtain the optimization problem function

6 $$\begin{aligned} L\left( p_i, \lambda , b\right) =f\left( p_i\right) +\sum _{i=1}^N \lambda _i\left( 1-p_i\right) +\sum _{j=1}^N \mu _i\left( p_j-B_j\right) +b\left( \sum _{k=1}^N p_k-P\right) , \end{aligned}$$

where $i \in [1,2, \ldots , N],\lambda _i$, $\mu _i$, *b* are the Lagrangian multipliers of the constraints, respectively.

### AMM liquidity mathematical model

The AMM introduced in this section is a centralized liquidity AMM for trading the entire NFT in a liquid cross-chain system, which means that the range of demand conditions for providing liquidity can be freely customized, i.e., the AMM mechanism is used to solve the liquidity problem of the NFT^31–33^. At the same time, AMM does not split NFT, but rather facilitates cross-chaining of full-chain NFT using a bonding curve and allows for unilateral liquidity pools of NFT or ETH, each managed by a liquidity demand, and the parameters of the liquidity pool are readily adjustable when NFT is paired with ETH. Thus, the conditions for cross-chaining are determined by the bonding curve style, facilitating the unique flow of NFT across the chain in a liquidity cross-chain system.

**Fig. 2:**
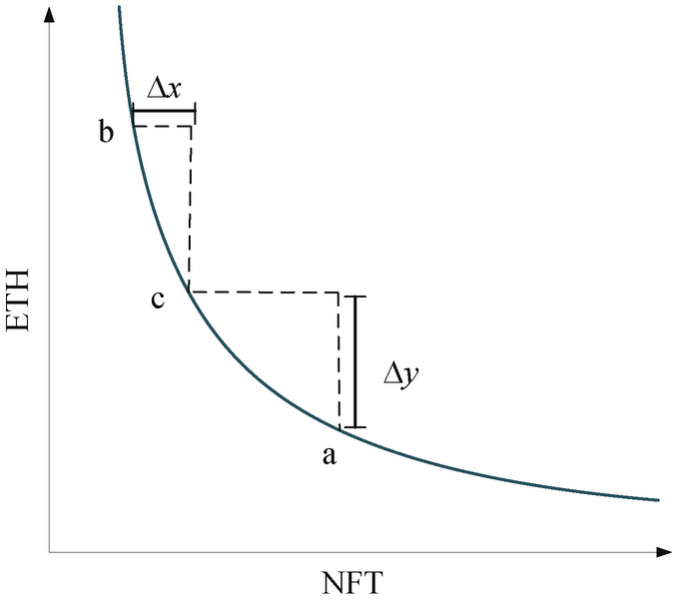
AMM function chart.

A traditional AMM allows providing liquidity, supporting infinite values, based on the function $x * y = k$, where *x*,*y* represent respectively the number of two assets in the liquidity pool and their product is a constant *k*, as shown in Fig. 2. A trading pair is based on two Tokens, let’s assume they are X and Y. Then *x* and *y* are the number of tokens X and Y have as reserves in this trading pair. This ratio of pairs is determined based on the number of X and Y in the pair. The *k* in $y * x = k$ is certain during the whole trading process. It is possible to keep the two coins to be traded at a corresponding price and quantity all the time. Set the trading formula as shown below.

7 $$\begin{aligned} xy=(x+\Delta x)(y-\Delta y), \end{aligned}$$

where $\Delta x$ represents the value-added coins and $\Delta y$ represents the sold coins, set $\alpha = \frac{{\Delta x}}{x}$ and $\beta = \frac{{\Delta y}}{y}$. The equations that can be derived are shown below.

8 $$\begin{aligned} x+\Delta x= & \frac{xy}{y-\Delta y}=\frac{1}{1-\beta }x, \end{aligned}$$

9 $$\begin{aligned} y-\Delta y= & (1-\beta )y, \end{aligned}$$

10 $$\begin{aligned} \Delta x= & \frac{\beta }{1-\beta }x, \end{aligned}$$

11 $$\begin{aligned} \Delta y= & \frac{\alpha }{1+\alpha }y. \end{aligned}$$

When the *k* of the model is not constant but will have a slow growth with the transaction, it shows the liquidity share calculation and updates to achieve the mathematical model of increasing liquidity across the chain of transactions, as shown in the following equation.

12 $$\begin{aligned} \left( e,t,l\right) \rightarrow \left( e^\prime ,t^\prime ,l^\prime \right) , \end{aligned}$$

where *e* represents the number of ether, *t* represents the number of transactions to another coin, and *l* represents the number of increased liquidity. $e^{'}$, $t^{'}$, $l^{'}$ represent the values after the state has changed, respectively, and are all at rest, that is, their values change only after changing from one state to the next, and let $\alpha = \frac{{\Delta e}}{e}$, that is

13 $$\begin{aligned} e^\prime= & (1+\alpha )e, \end{aligned}$$

14 $$\begin{aligned} t^\prime= & (1+\alpha )t, \end{aligned}$$

15 $$\begin{aligned} l^\prime= & (1+\alpha )l, \end{aligned}$$

where the ratio of *e* : *t* : *l* is fixed, i.e.

16 $$\begin{aligned} e:t:l=e^\prime :t^\prime :l^\prime . \end{aligned}$$

## Experiment results and analysis

This section describes the experimental environment, in which the underlying blockchain framework was built and four cross-chain systems were enabled. Confirming the configuration of the system and software for cross-chain communication in ERC-20 standard, Bulletproofs, Cosmos ERC-20 standard, and the proposed model in Ethernet, respectively. Firstly, the cross-chain data is analyzed based on observing the three characteristic performance indicators of the four models and the incentive detection of the proposed model, then the cross-chain model is analyzed based on the three observed characteristic performance indicators, and finally, the conclusions related to the mobility of the cross-chain model are summarised and analyzed by comparing the three characteristic performance indicators of the cross-chain model, i.e., the timeliness of the model, the robustness, and the radius of convergence of the cross-chain model.

### Experimental analysis

At the beginning of the study, 44 existing cross-chain bridge models were analyzed and compared. The summary analysis resulted in four categories of cross-chain bridge models, namely asset-centric cross-chain bridge models (category 5), special cross-chain bridge models (category 14), applied cross-chain bridge models (category 11), and generic cross-chain bridge models (category 14). Several liquidity cross-chain technologies have emerged in the market, such as the ERC-20 standard, Bulletproofs, and Cosmos ERC-20 standard in Ethernet, which are developed based on Ethernet and can be implemented and operated on Ethernet. In the liquidity blockchain cross-chain model, the time spent in the process of transferring or circulating value by some technical means is called cross-chain timeliness. Ten groups of cross-chain transaction data are set to be passed through four groups of liquidity cross-chain models, and the file sizes of each group are 50 B, 100 B, 150 B, 200 B, 250 B, 300 B, 350 B, and 400 B, and the experimental data are configuration data, account data, block data, transaction data, entity data, contract data, and the data that contain large files, confidential and sensitive data, and redundant and repetitive data. Finally, the length of time spent on cross-chain is counted.

### Test performances

In blockchain cross-chain transactions, the efficiency of cross-chain technology can be weighed in terms of its relevant performance metrics. Therefore, in this section, three important characteristic indicators in cross-chain transactions are analyzed to enhance the liquidity cross-chain model. The three important characteristic metrics in the liquidity cross-chain model are as follows.

**Fig. 3:**
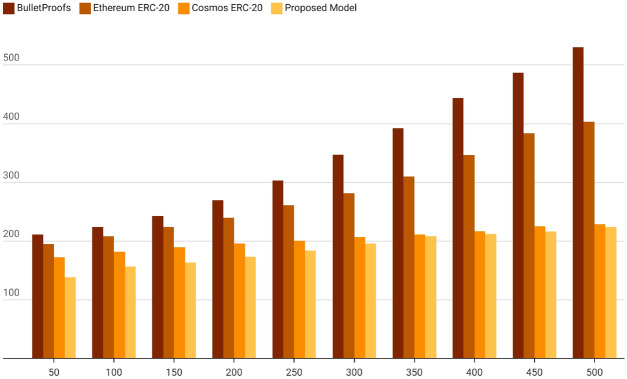
Time-consuming performance comparison.

*Performance measure 1* From the ten sets of experimental results, it can be concluded that the overall time consumption of the proposed model is much less than the other three sets of mobility cross-chain models. As the number of bytes increases, the proposed model shows more efficient and stable performance. When the number of bytes exceeds 350, the stability of the proposed model is especially outstanding, and it also shows that the impact of the increase in the number of bytes is within a certain controllable range. The results of the time-consuming comparison are shown in Fig. 3.

**Fig. 4:**
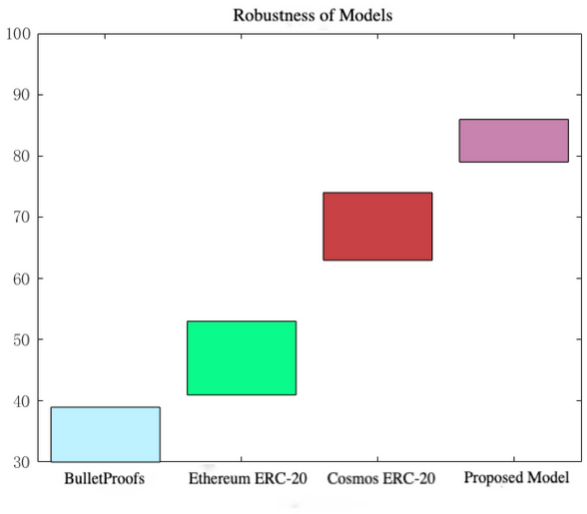
Robustness comparison chart.

*Performance measure 2* In the liquidity blockchain cross-chain model, the nodes of the blockchain system model are used as the point set, the block state is used as the line set, and the blockchain performance is used as the surface to define the robustness parameters of the consensus algorithm in the liquidity cross chain model. Then, the robustness parameters of the consensus algorithm in the liquidity cross-chain model are classified and analyzed, and finally, the initial design set is established to ensure the optimal operation of the liquidity cross-chain model. As shown in Fig. 4, the average robustness index of different sizes of data groups after 10 iterations in liquidity cross-chain interoperability is displayed. The robustness of the proposed model is much higher than the other three sets of data, indicating that the liquidity cross-chain model’s cross-chain rate changes will be adjusted accordingly with changes in the cross-chain demand dataset, and can resist a large number of errors, applause, and overload issues. Furthermore, it can be concluded that under the same circumstances, the current model has a very strong resistance to data transactions.

**Fig. 5:**
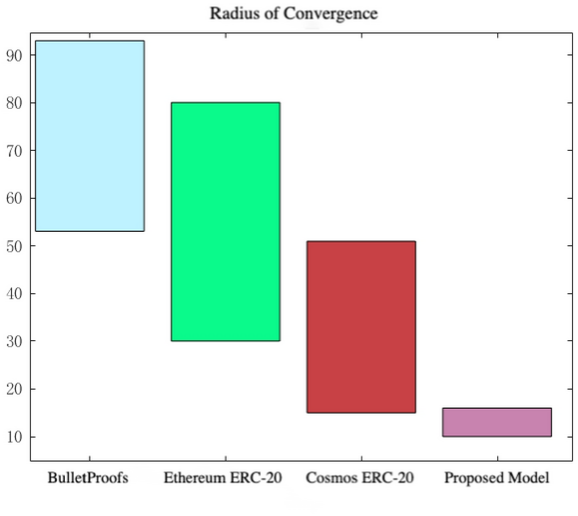
Radius of convergence comparison chart.

*Performance measure 3* As cross-chain transactions grow, to maintain liquidity, cross-chain models must continuously achieve a stable operating point. When cross-chain transactions approach a certain point, the convergence radius ensures their security and stability. Thus, a smaller convergence radius indicates greater model security and stability. As illustrated in Fig. 5, the convergence radius for different data set sizes, after iterations aiming for safety and stability in cross-chain model interoperability, is displayed. Observations of the model’s cross-chain iterations demonstrate that current technology ensures security and stability only for byte counts less than 150, whereas the proposed liquidity cross-chain model consistently operates stably. Consequently, the proposed model offers exceptional security and robust risk management.

### Incentives for liquidity cross-chain incentive detection

**Fig. 6:**
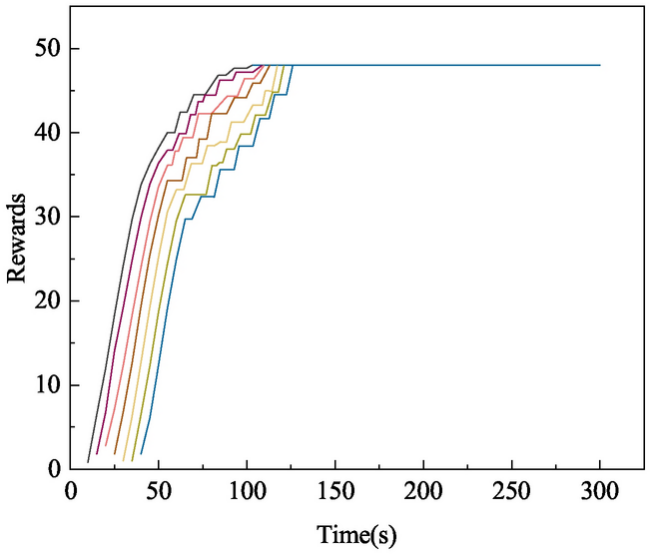
Multiple groups liquidity cross-chain reward time pairs.

In this paper, we design a convex optimization-based cross-chain model for liquidity contract pairs, both the number of cross-chains and the cross-chain expectation are determined in determining the convex optimized liquidity contract pairs. The number of cross-chains affects the cross-chain reward, and the reward also affects the probability of occurrence of each pair of cross-chains. As shown in Fig. 6 for multiple groups of liquid cross-chain reward time pairs.

When liquidity cross-chaining begins, each data set demonstrates a very active liquidity cross-chaining state as time progresses. However, as each round of cross-chaining passes, a delayed state occurs, where rewards grow in a folded pattern and no longer in a high-speed curved pattern, as the system sorts the contract pairs for reward evaluation. Liquidity cross-chaining continues only if no objection message is received. If an objection message is received, the system will pause the cross-chain. It is worth noting that when the number of cross-chaining is certain, the cross-chaining rewards and cross-chaining time have a close relationship, and at the same time, due to the system set up the additive reward mechanism and the final elimination mechanism, with the growth of time the greater the rewards, and finally it will reach a peak, and no longer have changed.

## Conclusion

### Model comparisons

To precisely and efficiently meet the different needs of users in asset trading, data sharing, and trustworthy deposition, researchers have mainly explored and researched the existing liquidity cross-chain technologies in terms of cryptography, naming protocols, authentication, and communication protocols, and cross-chain transaction consistency. In this paper, we propose a convex optimization-based liquidity contract for a cross-chain model and firstly, we examine the performance of the liquidity cross-chain model in terms of time consumption, robustness, and radius of model convergence. The cross-chain speed can be increased by 55.6%, the model robustness can be increased by up to 92.7%, and the model convergence radius can be reduced by up to 68.6%. Secondly, by using the cross-chain environment and cross-chain demand, the convex optimization mathematical model is used to first obtain the optimal liquidity contract pairs, and then determine the number of cross-chains and cross-chain expectation, while the number of cross-chains will determine the cross-chain reward and cross-chain time, and the cross-chain reward will grow with time, but will eventually reach a maximum value without any special change.

This area of liquidity cross-chaining technology is still in a stage of fundamental development and cannot rely on liquidity pools alone. If we want to achieve the free liquidity cross-chain approach, a single technical approach is still far from enough, which requires a complete system of innovation and development, in order to be able to achieve the true meaning of the integration between the blockchain, breaking the blockchain silo phenomenon.

### Limitations and future work

In this study, intelligent consensus mechanisms such as throughput and incentives in the model for the service chain need to be further improved^34,35^. Meanwhile, as an emerging technology in the digital asset sector, the circular liquidity cross-chain model has a broader outlook. With the continuous development of cryptocurrencies and liquidity cross-chain, the cross-chain model needs to further improve the privacy and security features to provide more options for digital asset holders. Multi-chain attributes and circular flow cross-chain models complement each other to provide users with more cross-chain transaction opportunities and promote the further development of the digital currency market.

In the future, the liquidity cross-chain model will serve as a foundation to assist the circular liquidity cross-chain model to play an important role in the digital currency market. Further research can be done to add incentives and private security mechanisms to the model in terms of optimizing the consensus mechanism to increase the throughput of the service chain.

## Data Availability

The experimental data for this study is publicly available and secure. Some models, or code that support the findings of this study are available from the author Yutong Han upon reasonable request via email.
